# Pathological Skin Picking: Phenomenology and Associations With Emotions, Self-Esteem, Body Image, and Subjective Physical Well-Being

**DOI:** 10.3389/fpsyt.2021.732717

**Published:** 2021-10-13

**Authors:** Christina Gallinat, Linda Luisa Stürmlinger, Sandra Schaber, Stephanie Bauer

**Affiliations:** ^1^Center for Psychotherapy Research, University Hospital Heidelberg, Heidelberg, Germany; ^2^Graduate School of Economic and Social Sciences (GESS) University of Mannheim, Mannheim, Germany

**Keywords:** skin picking disorder, excoriation disorder, phenomenology, emotions, self-esteem, body image, well-being

## Abstract

Pathological skin picking (PSP) refers to the repetitive manipulation of the skin causing wounds, scars, emotional distress, and social impairment. Skin picking disorder was first recognized as a distinct disorder in the DSM-5 and is still understudied in terms of phenomenology, etiology, and associated consequences. However, the body-related pathology suggests that the relation to the own body might be a crucial factor in PSP. Previous studies provided first insights that affected individuals show a more negative body image and lower self-esteem than healthy individuals. The present study sought to investigate skin picking phenomenology, related emotions as well as associations with self-esteem, body image, and subjective physical well-being. The study was conducted as an open online study, and recruitment was generally targeted to individuals of full age and in addition specifically to individuals with PSP. A total of 363 individuals (82.9% female; age: *M* = 32.6, *SD* = 13.92) participated and answered various self-report measures. Nearly half of the sample exceeded the cutoff score for pathological skin picking (*N* = 163; 44.9%). The results suggest that boredom, bodily tension and strong negative feelings often precede PSP episodes. During the episode most individuals seem to experience a loss of control, trance and positive feelings. After the PSP episode, shame, guilt, anger and anger toward themselves are most prominent. As expected, skin picking severity was positively associated with body image disturbances and negatively with self-esteem, and subjective physical well-being. When controlling for depressive symptoms, all associations were reduced, but those with body image disturbances (*r* = 0.44; *p* < 0.001), self-esteem (*r* = −0.27; *p* < 0.001), subjective physical well-being (*r* = −0.22; *p* < 0.001), and peace of mind (*r* = 0.30; *p* < 0.01) remained significant. Moreover, greater skin damage due to skin picking was moderately associated with higher body image disturbances. The results indicate that PSP severity is associated with a negative body image and low self-esteem, and suggest that it may be warranted to consider these aspects in the development of future interventions for PSP. However, relations with body image and self-esteem should be examined in longitudinal studies investigating causal relationships between body image, self-esteem and skin picking. Moreover, PSP phenomenology and the role of specific emotions should be investigated in more detail.

## Introduction

Pathological skin picking (PSP) is an uncontrollable, body-focused repetitive behavior (BFRB) of manipulating one's own skin, leading to skin damage and psychosocial impairment. The associated mental condition is called “excoriation (skin picking) disorder” and affects about 1.4% of the population during lifetime (1). In 2013, the disorder was recognized as a distinct diagnosis under the category of obsessive-compulsive and related disorders (1). Previous studies indicate high comorbidities especially with depression, anxiety disorders, trauma-related disorders, substance-related disorders, obsessive-compulsive disorder, and other disorders in the obsessive-compulsive spectrum including trichotillomania (2–4).

Individuals with skin picking disorder intensely pick their skin by e.g., squeezing, rubbing, and scratching. Most often fingernails, but also tweezers, needles and one's own teeth are used to manipulate the skin (5). The behavior usually affects multiple body areas and the most commonly affected skin regions are face, scalp, arms, and legs [e.g., (5–7)]. A large study with 760 participants reported an average of 4.73 affected body areas per person (5). The time spent on skin picking highly varies in and between individuals and can range between a few minutes and several hours per day. Tucker et al. (5) for example reported a mean number of 8.35 (*SD* = 21.65) skin picking episodes per day, which were on average 21 min long (*SD* = 42.24). However, deviating results from other studies underline the high variability in terms of time spent on the behavior as well as the need for further research [e.g., (6–8)].

Literature suggests that skin picking often occurs in reaction to negative emotions as it usually provides short-term relief of tension, but during and after the skin picking episode, negative states like shame, guilt, and pain seem to increase (6, 9, 10). These mechanisms often lead to a vicious cycle of initiating and prolonging episodes of skin picking behavior. Overall, a growing body of research suggests that PSP might serve as a maladaptive emotion regulation mechanism (11, 12). Although the mentioned studies provide important insight on these mechanisms, specific emotions related to PSP have yet to be investigated in more detail.

Individuals with skin picking disorder are unable to stop the behavior, even though it leads to tissue damage, scarring, and other medical risks (e.g., infections) (5). In addition to these physical consequences, PSP also leads to emotional distress, shame and self-reproaches. Due to feelings of shame and guilt, most sufferers try to cover affected body areas and avoid social situations, where coverage is not possible (7). This avoidance does not only lead to emotional strain and impairment in close relationship, but also in occupational contexts (7). Moreover, there is evidence that the quality of life in individuals with PSP is lower compared to individuals without PSP (3, 8).

A low self-esteem is also often discussed as a consequence of PSP [e.g., (13)]. As feelings of guilt and shame are known to be related to low self-esteem (14), it seems highly plausible to expect self-esteem problems in individuals with PSP. In addition, wounds and scars caused by the PSP also pose a challenge to self-worth, as they are not in line with the general beauty ideal of flawless skin. Moreover, in a study of Maraz et al. (15) a general grooming factor across different BFRBs was positively associated with contingent self-esteem, which refers to the perceived self-worth related to the extent to which one meets certain standards and expectations. Additionally, the contingent self-esteem seems to be negatively associated with general self-esteem (16). In other words, the more one's evaluation of oneself depends on others, the lower one's own self-esteem (16). According to this, the finding of Maraz et al. (15) indirectly suggests a lower self-esteem in individuals with BFRBs, including those with PSP. However, to the best of our knowledge, self-esteem in individuals with PSP has not been investigated in detail so far.

Another understudied aspect of self-evaluation is the body image of individuals with PSP. Body image refers to a multidimensional construct comprising emotions, perceptions and cognition about one's own body (17). There are multiple reasons why body image might play an important role in PSP: First, PSP is directly focused on one's own body and it may be assumed that the way the own body is perceived also influences how it is treated. Second, PSP leads to skin damage and often permanent scarring, which likely affects how somebody evaluates his or her body. Third, PSP related psychosocial impairment is usually driven by shame and the fear of how one's own body might be perceived and evaluated by others.

Previous studies also provide first hints toward a negative body image in individuals with PSP. For example, Odlaug et al. (18) reported that women and men with PSP perceived themselves as less attractive than individuals without PSP. In addition, the PSP group reported more days of poor physical health in the past 30 days than the control group. Another study analyzed body-esteem in adolescents with hair pulling and individuals with hair pulling and skin picking (19). The study found that the group with hair pulling and skin picking report significantly lower appearance-based body esteem compared to those with only hair pulling. Moreover, Solley and Turner (20) compared individuals with various BFRBs (skin picking, hair pulling, nail biting, lip/cheek biting or chewing) with controls without BFRBs and reported that those with BFRBs indicated significantly more body image concerns than the control group. The skin picking group also showed significantly greater levels of body image concerns compared to the other three specific BFRBs groups. Overall, literature suggests preliminary evidence that body image might be a crucial factor in PSP.

Despite this previous research, knowledge on the discussed aspects of skin picking disorder is still limited and most studies referring to the phenomenology of the disorder have been published more than 10 years ago. The inclusion of skin picking disorder in the DSM-5 clearly calls for sound research on the disorder and its treatment. In particular, the comprehensive investigation of skin picking phenomenology, underlying mechanisms as well as maintaining factors and consequences of PSP is essential for a better understanding of the disorder and ultimately for the development of effective interventions for PSP. Given this need for research, the aim of the present study was the investigation of central phenomenological aspects including emotions associated with PSP episodes, self-esteem, and body image with subjective physical well-being.

Based on previous studies, it was expected that skin picking would be associated with negative affect before and after the episodes and that trance and positive feelings would play a role during the episodes. Moreover, based on earlier results and due to the psychosocial and physical consequences of PSP, it was hypothesized that PSP severity would be negatively associated with self-esteem and subjective physical well-being, and positively associated with body image disturbances.

## Materials and Methods

### Procedures

The present study was conducted as an anonymous open online-study in May and June 2020. Participants were recruited via social media networks, skin picking support groups, and mail distribution at universities. The study call was addressed to individuals with and without skin picking symptomatology. To avoid selection bias, participants were told that the aim of the study was to investigate mechanisms contributing to mental health problems. Specifically, the information materials stated that the objective was to examine how individuals perceive their own bodies, feelings and certain behaviors (e.g., intense scratching of their own skin). Participants needed to provide informed consent and had to be at least 18 years old. No other inclusion criteria were applied. All study procedures were approved by the ethics committee of the Medical Faculty of Heidelberg University.

### Measures

#### Sociodemographics and Clinical Variables

Assessed demographic information included age, gender, weight, height, and occupational status. Participants were asked, if they were currently in professional treatment due to a mental health problem. If so, they were asked to specify the problem by selecting from a multiple choice list of psychiatric diagnoses.

#### Skin Picking Severity

The German version of the Skin Picking Scale-Revised [SPS-R; (21, 22)] was applied to assess skin picking severity. The SPS-R consists of eight items assessing symptom severity (#1 urge frequency, #2 urge intensity, #3 time spent on skin picking, #4 controllability) and impairment (#5 emotional distress, #6 interference, #7 avoidance, #8 skin damage caused by skin picking) in reference to the last week. All items are rated on a 5-point Likert scale from 0 (e.g., “none”) to 4 (e.g., “extreme”). The SPS-R demonstrated excellent internal consistencies for the total scale (α = 0.95) and the subscales (symptom severity: α = 0.94; impairment: α = 0.92) in the present study.

#### Skin Picking-Related Impairment

The German version of the Skin Picking Impact Scale [SPIS; (23, 24)] was used to assess skin picking-related impairment. The SPIS consists of ten items, which are rated on a 5-point Likert scale from 0 (“not at all”) to 4 (“severe”) and refer to the last week. In the present study, the scale demonstrated excellent internal consistency (α = 0.95). The instrument was only administered to individuals with a SPS-R score of seven or higher.

#### Skin Picking Phenomenology and Associated Emotions

Skin picking phenomenology was assessed with 36 additional questions regarding affected skin regions, frequency and duration of skin picking episodes, skin damage due to skin picking (wounds/scars), and emotions before, during and after skin picking episodes. Like the SPIS, this instrument was also only administered to individuals above the SPS-R cutoff score.

#### Self-Esteem

Self-esteem was assessed with the revised German version of the Rosenberg Self-Esteem Scale (25, 26). The scale has ten items, which are rated on a 4-point Likert scale from 0 (“does not apply at all”) to 3 (“totally applies”). Higher values indicate greater self-esteem. The scale demonstrated an excellent internal consistency in the present study (α = 0.92).

#### Body Image Disturbances

Body image disturbances and associated impairment were assessed with the German version of the Body Image Disturbance Questionnaire [BIDQ; (27, 28)]. The questionnaire consists of seven items, which are rated on a 5-point Likert scale from 1 (e.g., never) to 5 (“very often”). Higher values indicate higher body image concerns and related impairment. Five additional open questions assess details on the reported concerns and impairments. In the present study, only one open question was used to assess the specific concerns about the appearance of the body or body parts. The internal consistency of the BIDQ was excellent in the present study (α = 0.92).

#### Appearance Related Body Image Aspects

Appearance related aspects of the body image were assessed with the German version of the Multidimensional Body-Self Relations Questionnaire—Appearance Scales [MBSRQ-AS; (29, 30)]. The questionnaire comprises 34 items forming five subscales, which are rated on a 5-point Likert scale. In the present study, only the scales “appearance evaluation” (7 items), “appearance orientation” (12 items) and “overweight preoccupation” (4 items) were used. In the present sample, the subscales showed acceptable to very excellent internal consistencies (α = 0.78–91).

#### Subjective Physical Well-Being

The FEW-16 [Fragebogen zur Erfassung der körperlichen Wohlbefindens; Questionnaire for Assessing Subjecitve Physical Well-being; (31)] was used to assess subjective physical well-being with the four dimensions “resilience,” “vitality,” “ability to enjoy,” “peace of mind.” All 16 items are rated on a 6-point Likert scale from 0 (“does not apply”) to 5 (“totally applies”) and refer to the last 3 weeks. Higher scores indicate higher subjective physical well-being. In the present study, the questionnaire showed good to excellent internal consistencies (total scale: α = 0.94; subscales: α = 0.87–91).

#### Depressive Symptoms

The Patient Health Questionnaire-9 [PHQ-9; (32)] was used to assess depressive symptoms. The PHQ-9 consists of nine items, which refer to the last 2 weeks. All items are rated on a 5-point Likert scale from 0 (“not at all”) to 4 (“almost every day”). Higher scores indicate a higher symptom level. In the present study, the PHQ-9 showed a good internal consistency (α = 0.88).

#### Weight Concerns

Weight concerns and cognitive preoccupation with weight and shape were assessed with the German version of the Weight Concerns Scale consisting of five items (33). Scores can range between 0 and 100, and higher scores indicate more preoccupation with and concern about weight and shape. A score above 57 is assumed to indicate a high risk for eating disorders (33, 34). In this study, a good internal consistency of the questionnaire was found (α = 0.80).

### Statistical Analysis

Data on skin picking phenomenology were analyzed by means of descriptive statistics. Continuous variables regarding emotions before, during and after PSP were dichotomized by splitting 4-point Likert scales in the middle (e.g., disagree: “never,” “rarely”; agree: “sometimes,” “always”).

Intrapersonal differences between emotions during and after PSP episodes were analyzed by paired sample *t*-tests. Pearson product moment correlations were used to analyze associations between skin picking severity, self-esteem, body image and subjective physical well-being, and associations between body image disturbances and skin damage (scars/wounds due to skin picking). In addition, partial correlations were calculated to control for depressive symptoms.

Data analyses were conducted with IBM® SPSS® Statistics 25.0 (SPSS, Inc., Chicago, IL).

## Results

### Participants

In total, 460 individuals started the online questionnaire, and 364 completed the assessment. One person had to be excluded, due to being underage. The final sample consisted of 363 individuals of whom 163 (44.9%) exceeded the cutoff score for pathological skin picking (PSP; SPS-R total score ≥ 7). The majority of the sample was female (82.9%) and age ranged between 18 and 80 (*M* = 32.64, *SD* = 13.92). Most participants were employed (43.5%) or college students (35.5%).

Overall, 57% (*N* = 207) of the total sample reported being currently affected by mental health problems, and 27.6% (*N* = 45) in the PSP group and 7.5% (*N* = 15) in the non-PSP group were currently in professional treatment due to a psychological problem. Most commonly mentioned mental conditions in both groups were depressive disorders and anxiety disorders, and additionally skin picking disorder in the PSP group. The PHQ-9 scores indicated moderate depressive symptoms in the PSP group (*M* = 10.37, *SD* = 6.00) and mild depressive symptoms in the non-PSP group (*M* = 5.64, *SD* = 4.19).

In terms of weight concerns, the PSP group scored higher overall and 20.9% (*N* = 34) were above the cutoff of 57 for being at risk for eating disorders, compared to only 9.5% (*N* = 19) in the non-PSP group. Demographic and clinical variables are presented separately for each group in Table 1.

**Table 1:** Demographic characteristics and clinical variables.

	**Total**	**PSP group**	**No PSP**
	**(*N* = 363)**	**(*N* = 163)**	**(*N* = 200)**
Gender			
Female	82.9% (*N* = 301)	93.3% (*N* = 152)	74.5% (*N* = 149)
Male	16.8% (*N* = 61)	6.1% (*N* = 10)	25.5% (*N* = 51)
Diverse	0.3% (*N* = 1)	0.6% (*N* = 1)	/
Age^a^	32.64 (13.92)	28.94 (9.29)	35.65 (16.19)
BMI^a^	23.94 (5.28)	23.25 (5.34)	24.49 (5.17)
SPS-R Total score^a^	7.83 (7.52)	15.00 (5.24)	1.99 (2.06)
SPS-R Symptom severity^a^	5.01 (4.33)	9.15 (2.67)	1.65 (1.69)
SPS-R Impairment^a^	6.82 (3.52)	5.85 (3.22)	0.35 (0.62)
SPIS^a^	/	17.46 (11.93)	/
PHQ-9^a^	7.77 (5.59)	10.37 (6.00)	5.64 (4.19)
WCS^a^	32.59 (21.90)	37.56 (23.99)	28.53 (19.16)
Self-reported mental health problems^b^			
Skin picking disorder	25.4% (*N* = 92)	56.4% (*N* = 92)	/
Depressive disorders	21.5% (*N* = 78)	35.0% (*N* = 57)	10.5% (*N* = 21)
Anxiety disorders	14.6% (*N* = 53)	23.3% (*N* = 38)	7.5% (*N* = 15)
Trauma related disorder	8.0% (*N* = 29)	11.0% (*N* = 18)	5.5% (*N* = 11)
Eating disorder	5.5% (*N* = 20)	8.0% (*N* = 13)	3.5% (*N* = 7)
Obsessive-compulsive disorder	4.4% (*N* = 16)	9.2% (*N* = 15)	0.5% (*N* = 1)
Personality disorder	4.4% (*N* = 16)	6.1% (*N* = 10)	3.0% (*N* = 6)
Impulse control disorder	3.9% (*N* = 14)	8.0% (*N* = 13)	0.5% (*N* = 1)
ADHD	3.3% (*N* = 12)	4.9% (*N* = 8)	2.0% (*N* = 4)
Body dysmorphic disorder	2.2% (*N* = 8)	4.9% (*N* = 8)	/
Adjustment disorder	1.7% (*N* = 6)	2.5% (*N* = 4)	1.0% (*N* = 2)
Dissociative disorder	1.7% (*N* = 6)	3.1% (*N* = 5)	0.5% (*N* = 1)
Substance use disorder	1.1% (*N* = 4)	2.5% (*N* = 4)	/
Trichotillomania	1.1% (*N* = 4)	2.5% (*N* = 4)	0.5% (*N* = 1)
Bipolar disorder	0.8% (*N* = 3)	1.8% (*N* = 3)	/
Schizophrenia or psychotic disorder	0.6% (*N* = 2)	1.8% (*N* = 3)	/
Other	2.5% (*N* = 9)	3.6% (*N* = 6)	1.5% (*N* = 3)
“Not sure”	13.2% (*N* = 48)	14.7% (*N* = 24)	12.0% (*N* = 24)
“Not willing to indicate”	0.6% (*N* = 2)	14.7% (*N* = 24)	1.0% (*N* = 2)
Any mental health problem	57.0% (*N* = 207)	85.9% (*N* = 140)	33.5% (*N* = 67)

a *M(SD).*

b *Participants could select multiple mental health problems. PSP, pathological skin picking (SPS-R total score ≥ 7); SPS-R, Skin Picking Scale-Revised; SPIS, Skin Picking Impact scale; PHQ-9, Patient Health Questionnaire; WCS, Weight Concerns Scale*.

### Skin Picking Phenomenology

Participants had suffered from PSP for an average 13.81 years (*SD* = 9.37) at the time the study was conducted, and the majority reported PSP at almost every day or several times per day (71.7%; *N* = 114). Moreover, most participants indicated one to five episodes per day (78.0%; *N* = 124), which were mostly between 1 and 30 min long (75.5%; *N* = 120). Periods without skin picking in the last 12 months were no longer than 7 days for most participants (86.2%; *N* = 137). The majority reported three or more affected body areas (70.0%; *N* = 112), with face, chest and shoulders being mentioned most often (50–84.4%). In terms of tissue damage, most participants reported a mild to moderate severity of current wounds (82.9%; *N* = 131) and scars (72.7%; *N* = 115). But a minority also reported severe to extreme wounds (8.2%; *N* = 13) and scars (16.5%; *N* = 26). Table 2 provides all frequencies.

**Table 2:** Skin picking phenomenology.

	**% (*N*)**		**% (*N*)**
**Number of affected body areas**		**PSP frequency in the last 6 weeks**	
0	0.6 (1)	Rarely (1–2 times)	2.5 (4)
1	12.5 (20)	Sometimes (weekly)	8.2 (13)
2	16.9 (27)	Frequently (several times a week)	17.6 (28)
3	18.1 (29)	Almost every day	26.4 (42)
>3	47.5 (76)	Several times per day	45.3 (72)
All	4.4 (7)	**Typical episode frequency in last 2 weeks per day**	
**Affected body areas** ^**a**^		0	4.4 (7)
Face	84.4 (135)	1–5	78.0 (124)
Chest	55.0 (88)	6–10	11.9 (19)
Shoulders	50.0 (80)	11–20	4.4 (7)
Arms	43.1 (69)	>20	1.3 (2)
Back	41.3 (66)	**Typical episode duration**	
Legs	39.4 (63)	<1 min	7.5 (12)
Neck	33.8 (54)	1–5 min	35.2 (56)
Scalp	33.1 (53)	6–30 min	40.3 (64)
Hands	25.0 (40)	31–60 min	10.7 (17)
Belly	15.6 (25)	61–120 min	5.7 (9)
Feet	15.0 (24)	121–360 min	0.6 (1)
**Current wounds**		**Longest period without PSP in the last 12 months**	
None	8.9 (14)	<1 days	25.8 (41)
Mild (only few wounds)	41.8 (66)	1–3 days	34.6 (55)
Moderate (many wounds)	41.1 (65)	4–7 days	25.8 (41)
Severe (very many)	7.6 (12)	Several weeks	9.4 (15)
Extreme (wounds cover large areas)	0.6 (1)	Several months	4.4 (7)
**Current scars**			
None	10.8 (17)		
Mild (only few scars)	37.3 (59)		
Moderate (many scars)	35.4 (56)		
Severe (very many scars)	14.6 (23)		
Extreme (almost all areas are scarred)	1.9 (3)		

*N = 163 (up to 5 missing values); PSP, Pathological skin picking.*

a *Participants could select multiple affected body areas.*

*PSP, Pathological skin picking*.

### Emotions

About two-thirds of the affected individuals generally agreed that intense feelings trigger their skin picking behavior strongly or very strongly (58.9%; *N* = 93). When asked for specific emotional states, the majority of participants reported that PSP starts when they are experiencing boredom (86.7; *N* = 137), bodily tension (82.3; *N* = 130), and strong negative feelings (76.0; *N* = 120). During PSP, most participants experience a loss of control (72.2%; *N* = 114), trance (68.4%; *N* = 108), and positive feelings (69.0%; *N* = 109). About 50–60% also experience tension, shame, anger toward themselves, guilt, and relaxation. Referring to the time after the PSP episode, anger (77.2%; *N* = 122), shame (70.9%; *N* = 112), anger toward themselves (69.6%; *N* = 110), and guilt (68.4%; *N* = 108) are reported by the majority of the sample.

Paired sample *t*-tests comparing emotion scores during and after PSP showed statistically significant average differences in relaxation [*t*_(157)_ = 6.61, *p* < 0.001], guilt [*t*_(157)_ = −5.31, *p* < 0.001], shame [*t*_(157)_ = −6.22, *p* < 0.001], and anger toward oneself [*t*_(157)_ = −5.19, *p* < 0.001]. Relaxation decreases, while all other emotions increase after PSP. No significant differences in tension and pain were found. Details are presented in Table 3.

**Table 3:** Pathological skin picking and associated emotions.

	**% (*N*)**
**I pick my skin when I experience…**	
strong negative feelings (e.g., anxiety, anger, sadness)	76.0 (120)
strong positive feelings (e.g., joy, excitement)	26.0 (41)
Boredom	86.7 (137)
Bodily tension	82.3 (130)
Relaxation	29.1 (46)
**During skin picking I experience…**	
Positive feelings (e.g., satisfaction, joy)	69.0 (109)
Negative feelings (e.g., anxiety, anger, sadness)	36.7 (58)
Relaxation	58.2 (92)
Tension	53.2 (84)
Trance	68.4 (108)
Loss of control	72.2 (114)
Guilt	54.4 (86)
Shame	51.3 (81)
Anger toward myself	51.3 (81)
Pain	32.3 (51)
**After I picked my skin I experience…**	
Satisfaction	26.0 (41)
Relaxation	30.4 (48)
Tension	51.3 (81)
Anger	77.2 (122)
Anger toward myself	69.6 (110)
Sadness	55.7 (88)
Anxiety	20.9 (33)
Guilt	68.4 (108)
Shame	70.9 (112)
Pain	32.3 (51)

*N = 158; Answers on the 4-point Likert scale were dichotomized (Disagree: “does not apply at all,” “barely”/“never,” “rarely”; Agree: “applies somewhat,” “totally applies”/“sometimes,” “always”)*.

### Self-Esteem and Body Image

Skin picking severity showed a high negative correlation with self-esteem (*r* = −0.55; *p* < 0.001), as well as with subjective physical well-being (total score and subscales; *r* = −0.36 to −0.54; *p* < 0.001) and appearance evaluation (*r* = −0.32; *p* < 0.001). In contrast, skin picking severity was strongly positively correlated with body image disturbances (*r* = 0.62; *p* < 0.001), appearance orientation (*r* = 0.34; *p* < 0.001), and depressive symptoms (*r* = 53; *p* < 0.001).

Given the high correlations between all variables and depressive symptoms, partial correlations were calculated. After controlling for depressive symptoms, the correlations between skin picking severity and FEW-subscales resilience, vitality, appearance evaluation, and overweight preoccupation were near null. However, skin picking severity still showed small to medium associations with appearance orientation (*r* = 0.21; *p* < 0.001), as well as with the physical well-being total score (*r* = −0.22; *p* < 0.001), the subscales peace of mind (*r* = −0.30; *p* < 0.01), and ability to enjoy (*r* = −0.16; *p* < 0.01). Furthermore, skin picking severity still showed medium to strong correlations with body image disturbances (*r* = 0.44; *p* < 0.001) and self-esteem (*r* = −0.27; *p* < 0.001). Details are presented in Table 4.

**Table 4:** Associations between skin picking severity, self-esteem, body image, and physical well-being.

	**1**	**2**	**3**	**4**	**5**	**6**	**7**	**8**	**9**	**10**	**11**	**PHQ**
1 SPS-R	1	−0.55^a^	0.62^a^	−0.53^a^	−0.36^a^	−0.38^a^	−0.45^a^	−0.54^a^	−0.32^a^	0.34^a^	0.16^b^	0.53^a^
2 RSES	−0.27^a^	1	−0.67^a^	0.76^a^	0.51^a^	0.60^a^	0.70^a^	0.72^a^	0.66^a^	−0.36^a^	−0.33^a^	−0.76^a^
3 BIDQ	0.44^a^	−0.36^a^	1	−0.63^a^	−0.44^a^	−0.50^a^	−0.58^a^	−0.56^a^	−0.54^a^	0.45^a^	0.38^a^	0.65^a^
4 FEW total	−0.22^a^	0.43^a^	−0.27^a^	1	0.79^a^	0.82^a^	0.85^a^	0.86^a^	0.64^a^	−0.28^a^	−0.29^a^	−0.78^a^
5 FEW resilience	−0.08	0.15^b^	−0.11^c^	0.67^a^	1	0.50^a^	0.57^a^	0.58^a^	0.48^a^	−0.19^a^	−0.15^b^	−0.57^a^
6 FEW vitality	−0.05	0.19^a^	−0.12^c^	0.65^a^	0.20^a^	1	0.60^a^	0.61^a^	0.46^a^	−0.20^a^	−0.23^a^	−0.67^a^
7 FEW ability to enjoy	−0.16^b^	0.44^a^	−0.28^a^	0.71^a^	0.32^a^	0.29^a^	1	0.65^a^	0.68^a^	−0.23^a^	−0.32^a^	−0.66^a^
8 FEW peace of mind	−0.30^a^	0.44^a^	−0.22^a^	0.72^a^	0.32^a^	0.29^a^	0.36^a^	1	0.52^a^	−0.34^a^	−0.27^a^	−0.68^a^
9 MBSRQ-AS: Appearance evaluation	−0.04	0.44^a^	−0.30^a^	0.41^a^	0.25^a^	0.15^b^	0.51^a^	0.24^a^	1	−0.20^a^	−0.44^a^	−0.56^a^
10 MBSRQ-AS: Appearance orientation	0.21^a^	−0.19^a^	0.33^a^	−0.07	−0.01	0.02	−0.02	−0.17^b^	−0.03	1	0.35^a^	0.32^a^
11 MBSRQ-AS: Overweight preoccupation	−0.01	−0.17^b^	0.26^a^	−0.11^c^	0.02	−0.05	−0.18^b^	−0.09	−0.35^a^	0.28^a^	1	0.30^a^

*N = 355; SPS-R, Skin Picking Scale-Revised; RSES, Rosenberg Self Esteem Scale; BIDQ, Body Image Disturbance Questionnaire; FEW, Questionnaire for assessing subjective physical well-being; MBSRQ-AS, Multidimensional Body-Self Relations Questionnaire – Appearance Scales; PHQ-9, Patient Health Questionnaire-9 (depressive symptoms). Partial Correlations controlling for depressive symptoms are listed in the grey triangle.*

a *p < 0.001;*

b *p < 0.01,*

c *p < 0.05*.

Within the PSP subsample (*N* = 163), skin picking related impairment [assessed via SPIS (23)] showed strong associations with body image disturbances (*r* = 0.70; *p* < 0.001) and self-esteem (*r* = −0.42; *p* < 0.001). After controlling for depressive symptoms, the association with self-esteem (*r* = −0.25; *p* < 0.001) declined substantially, while the correlation with body image disturbances decreased only marginally (*r* = 0.65; *p* < 0.001).

### Body Image Disturbances and Skin Damage

In response to the open question in the BIDQ, 133 individuals clarified specific concerns about their bodies. Concerns about the skin were reported by 55.1% (*N* = 74) of this subsample. Besides that, primarily weight and shape were mentioned as sources of concern (48%; *N* = 64).

In the subsample of individuals with PSP (*N* = 163), body image disturbances were positively correlated with self-reported severity of wounds (*r* = 0.42; *p* < 0.001) and scars (*r* = 0.34; *p* < 0.001) due to skin picking. After controlling for depressive symptoms, both correlations were reduced, but remained significant (wounds: *r* = 0.31, *p* < 0.001; scars: *r* = 0.22, *p* < 0.01).

## Discussion

Pathological skin picking is an uncontrollable behavior that is strongly integrated into the daily life of affected individuals and causes significant psychosocial impairment and skin damage. However, the phenomenology and mechanisms of the behavior as well as its impact on self-esteem, body image and subjective physical well-being are still poorly understood. The present study investigated phenomenological parameters and associated emotions, and is to the best of our knowledge the first study analyzing self-esteem, subjective physical well-being and multiple aspects of body image in PSP in more detail.

In terms of phenomenology, the findings on the number and type of most affected skin regions are in line with former research and confirm that most individuals pick their skin at multiple body areas (5). Moreover, the results indicate that PSP occurs very frequently with the majority of participants reporting that they experience episodes almost every day or several times per day. Typically, episodes are of rather short to medium-duration (1–30 min) and occur one to five times a day. In addition, PSP free periods of more than 7 days in the last year were reported rarely.

The results regarding emotions related to PSP episodes confirm previous findings that negative affective states play an important role prior to PSP (9). Boredom, tension and negative affect seem to be prominent before an episode begins, while positive feelings (e.g., satisfaction), loss of control, and trance are present during the episode. After an episode, anger and anger toward oneself as well as guilt and shame are most prevalent. Compared to the results of Snorrason et al. (9) boredom and tension prior to PSP, positive feelings and trance during PSP, as well as guilt and shame after PSP were reported more often in the present study.

As expected, the present results indicate that skin picking severity is associated with low self-esteem and with body image disturbances, even after controlling for depressive impairment. So with higher PSP symptom severity, individuals experience more body image disturbances and lower self-esteem. Moreover, more severe skin damage due to skin picking was moderately associated with higher body image disturbances. This may be seen as a first indication that body image disturbances might be a direct consequence of PSP and its physical consequences. Of note, after controlling for depressive symptoms, skin picking severity was not associated anymore with appearance evaluation, overweight preoccupation and the FEW-16 (subjective physical well-being) subscales resilience and vitality.

However, independent of depressive symptoms skin picking severity showed significant small correlations with a higher appearance orientation (item example: “I am always trying to improve my appearance.”) and with a lower subjective physical well-being. Especially the FEW-16 subscale “peace of mind” (item example: “I am calm and relaxed.”) contributed to the latter association. The negative correlation with “peace of mind” also fits the clinical picture of PSP, which includes restless and wandering hands, which presumably also express inner restlessness.

Overall, the findings suggest that individuals with PSP suffer from a lower self-esteem and experience body image disturbances independent of depressive symptoms. The association between skin damage and body image disturbances may be a first hint that PSP caused skin damage may contribute to body image disturbances. However, beyond that, the study cannot make any statement about the causality of this relationship. This is also one of the limitations of the present study. Due to the cross-sectional study design, the analyses cannot clarify, if low self-esteem and high body image disturbances are consequences of PSP or perhaps also factors contributing to the pathogenesis of the disorder. Moreover, the assessed body image variables cover only a part of the multidimensional body image construct and can thus reflect only a part of these complex relationships. Consequently, body image in PSP should be investigated in more detail in future longitudinal studies.

It is also important to note that the many individuals in the PSP group reported comorbid mental health problems, particularly depression and anxiety, but also trauma related disorders, obsessive-compulsive disorder, eating disorders, impulse control disorders, and others. Since these disorders are also known to be associated with aspects like self-esteem and body image, future studies should take these into account when analyzing the associations with PSP in more depth.

The retrospective focus of the present study also needs to be considered as a limitation. This is especially relevant for the data on PSP phenomenology (e.g., duration/frequency of episodes) and associated emotions due to three reasons: First, PSP also often starts or occurs completely unconsciously. Second, PSP can highly vary within persons so that a retrospective estimation of the assessed parameters is inevitably imprecise to some extent. Third, emotions before, during and after PSP episodes are probably difficult to distinguish in the retrospective, so that the results need to be interpreted cautiously. In the light on these limitations, future studies should apply ecological momentary assessment to investigate PSP phenomenology and the role emotions in the daily life of affected individuals. This approach is particularly promising as it allows studying the variability of PSP, underlying temporal patterns and dynamic processes (35).

Other limitations result from the assessment via online self-report. The classification in PSP and non-PSP was based on a SPS-R cutoff score, which has already proven its validity in previous research (36), but the data do not refer to a clinical sample of individuals with skin picking disorder diagnosed with standard clinical interviews. Moreover, due to the online-setting it was not possible to control external interferences during assessment or to prevent individuals from participating more than once. Despite these limitations, the present study provides important insights in the phenomenology of PSP and its associations with certain emotions, self-esteem, body image and subjective physical well-being.

Overall, the results provide evidence of a strong association between PSP severity and body image disturbances and support the idea that body image problems, but also self-esteem are important aspects to include in therapeutic interventions. Moreover, the data on emotions associated with PSP episodes show, that shame, guilt and anger toward oneself play an important role in PSP. Against this background, interventions focusing on self- and body acceptance and self-compassion might also be promising starting points for comprehensive interventions for PSP and likely also other BFRBs like hair pulling. Given the lack of specific interventions for PSP, partly also caused by the paucity of basic research, the present data on body image and self-esteem but also on the phenomenology provide an important basis for future studies.

## Data Availability Statement

The datasets presented in this article are not readily available because of legal and privacy issues. Requests to access the datasets should be directed to Christina Gallinat (christina.gallinat@med.uni-heidelberg.de).

## Ethics Statement

The studies involving human participants were reviewed and approved by Ethics Committee of the Medical Faculty of Heidelberg University, Alte Glockengießerei 11/1, 69115 Heidelberg. The patients/participants provided their written informed consent to participate in this study.

## Author Contributions

CG coordinated and conducted the present study including conception, recruitment, as well as analysis and interpretation of data, drafted the manuscript, and approved the final version. LS contributed to the conception of the study, recruitment, analysis and interpretation of data, and revision of the article and final approval of the article. SS participated in the design of the study, recruitment and helped to revise the article, and approved the final manuscript. SB contributed to the conception and realization of the present study, as well as to the interpretation of data, revised the article critically, and approved the final version. All authors contributed to the article and approved the submitted version.

## Conflict of Interest

The authors declare that the research was conducted in the absence of any commercial or financial relationships that could be construed as a potential conflict of interest.

## Publisher's Note

All claims expressed in this article are solely those of the authors and do not necessarily represent those of their affiliated organizations, or those of the publisher, the editors and the reviewers. Any product that may be evaluated in this article, or claim that may be made by its manufacturer, is not guaranteed or endorsed by the publisher.
